# Anorexia Reduces GFAP+ Cell Density in the Rat Hippocampus

**DOI:** 10.1155/2016/2426413

**Published:** 2016-08-07

**Authors:** Daniel Reyes-Haro, Francisco Emmanuel Labrada-Moncada, Durairaj Ragu Varman, Janina Krüger, Teresa Morales, Ricardo Miledi, Ataúlfo Martínez-Torres

**Affiliations:** Departamento de Neurobiología Celular y Molecular, Instituto de Neurobiología, Universidad Nacional Autónoma de México, Campus Juriquilla, Boulevard Universitario 3001, 76230 Juriquilla, QRO, Mexico

## Abstract

Anorexia nervosa is an eating disorder observed primarily in young women. The neurobiology of the disorder is unknown but recently magnetic resonance imaging showed a volume reduction of the hippocampus in anorexic patients. Dehydration-induced anorexia (DIA) is a murine model that mimics core features of this disorder, including severe weight loss due to voluntary reduction in food intake. The energy supply to the brain is mediated by astrocytes, but whether their density is compromised by anorexia is unknown. Thus, the aim of this study was to estimate GFAP+ cell density in the main regions of the hippocampus (CA1, CA2, CA3, and dentate gyrus) in the DIA model. Our results showed that GFAP+ cell density was significantly reduced (~20%) in all regions of the hippocampus, except in CA1. Interestingly, DIA significantly reduced the GFAP+ cells/nuclei ratio in CA2 (−23%) and dentate gyrus (−48%). The reduction of GFAP+ cell density was in agreement with a lower expression of GFAP protein. Additionally, anorexia increased the expression of the intermediate filaments vimentin and nestin. Accordingly, anorexia increased the number of reactive astrocytes in CA2 and dentate gyrus more than twofold. We conclude that anorexia reduces the hippocampal GFAP+ cell density and increases vimentin and nestin expression.

## 1. Introduction

Anorexia nervosa is an eating disorder characterized by excessively restricted caloric intake that induces profound weight loss, osteoporosis, and amenorrhea [1]. The onset of anorexia nervosa is commonly observed during puberty and adolescence, with 90–95% of the cases occurring among females [2].

Due to its complexity, the neurobiology of anorexia nervosa is unknown, but studies using magnetic resonance imaging showed hippocampal volume reduction [3–6]. The hippocampus is involved in spatial learning, cognition, and regulation of anxiety [7]. Alterations in these cognitive functions have been reported in patients and in experimental models of anorexia. Murine models of anorexia such as dehydration-induced anorexia (DIA) or activity-based anorexia (ABA) mimic the characteristic weight loss and reduced food intake observed in anorexic patients [1, 2, 8–13]. The smaller hippocampal volume in anorexic patients may reflect structural changes at the cellular level. In the ABA model, changes in the hippocampus included reduced cell proliferation in the dentate gyrus and decreased branching of dendrites in the stratum radiatum of CA1 [1, 11, 12]. Excessive physical activity alters food ingestion, which can result in nutrient and electrolyte imbalance. Thus, alterations in hippocampal function and structure could be the result of reduced caloric intake that compromises the supply of energy to the brain. Astrocytes, one of the major populations of cells in the central nervous system, play a crucial role in supplying energy to neurons; therefore, we tested if hippocampal astrocyte density and intermediate filament expression are affected by anorexia.

## 2. Materials and Methods

### 2.1. Animals and Housing

The Institutional Animal Care and Use of Laboratory Animals Committee of the UNAM Instituto de Neurobiología approved all the experimental protocols. Animals were handled in accordance with the National Institute of Health Guide for the Care and Use of Laboratory Animals. Wistar female rats (160–190 g) were housed individually under 12-h/12-h light/dark cycle and controlled temperature, with food and water* ad libitum*.

### 2.2. Dehydration-Induced Anorexia

The protocol was performed as previously described [8, 13, 14]. Briefly, two independent experimental series of twelve animals were placed in individual cages; for each series, animals were randomly selected to form three groups of four. The first group received water and food* ad libitum* (control). The DIA group received a 2.5% NaCl solution as their sole drinking liquid and had unrestricted access to food. The forced food-restricted (FFR) group, a positive control to distinguish between starvation and dehydration effects, received tap water* ad libitum* and the same amount of food consumed by DIA animals. The experimental protocol was conducted for five days and body weight and solid food intake were recorded daily at noon for each experimental group. The FFR group received the same amount of food as that ingested by DIA animals (see Supplemental Figure 1 in Supplementary Material available online at http://dx.doi.org/10.1155/2016/2426413).

### 2.3. Histology

Rats were deeply anaesthetized with an overdose of sodium pentobarbital (100 mg/Kg) and transcardially perfused with 100 mL of saline followed by 250 mL of chilled 4% paraformaldehyde in phosphate-buffered saline (PBS) (pH 7.4). Brains were removed, postfixed overnight, and then transferred to a series of sucrose solutions (from 10 to 30%). Coronal sections (30 *μ*m) including the dorsal hippocampus were obtained on a freezing microtome, collected, and stored in cryoprotectant solution (30% ethylene glycol/20% glycerol in PBS) at −20°C [13].

### 2.4. Immunohistofluorescence

Glial fibrillary acidic protein (GFAP) immunoreactivity was performed on floating sections [13]. Briefly, coronal sections were rinsed three times in PBS buffer and treated with 3% hydrogen peroxide for 10 min, followed by three rinses in PBS and incubation in 1.0% sodium borohydride for 6–8 min to reduce free aldehydes. The sections were incubated for 1 h in blocking solution (5% horse serum albumin/1% Triton X-100 in PBS). Sections were incubated for 48 h with polyclonal rabbit anti-GFAP antibody (dilution 1 : 1000, DakoCytomation, Fort Collins, CO, USA; 4°C). After washing, primary antibody was detected with Alexa 594 (1 : 500, Invitrogen) coupled goat anti-rabbit secondary antibody. The sections were counterstained with 4′,6-diamidino-2-phenylindole (DAPI) and mounted with Vectashield H-1000 (Vector Laboratories, Burlingame, CA, USA).

**Figure 1:**
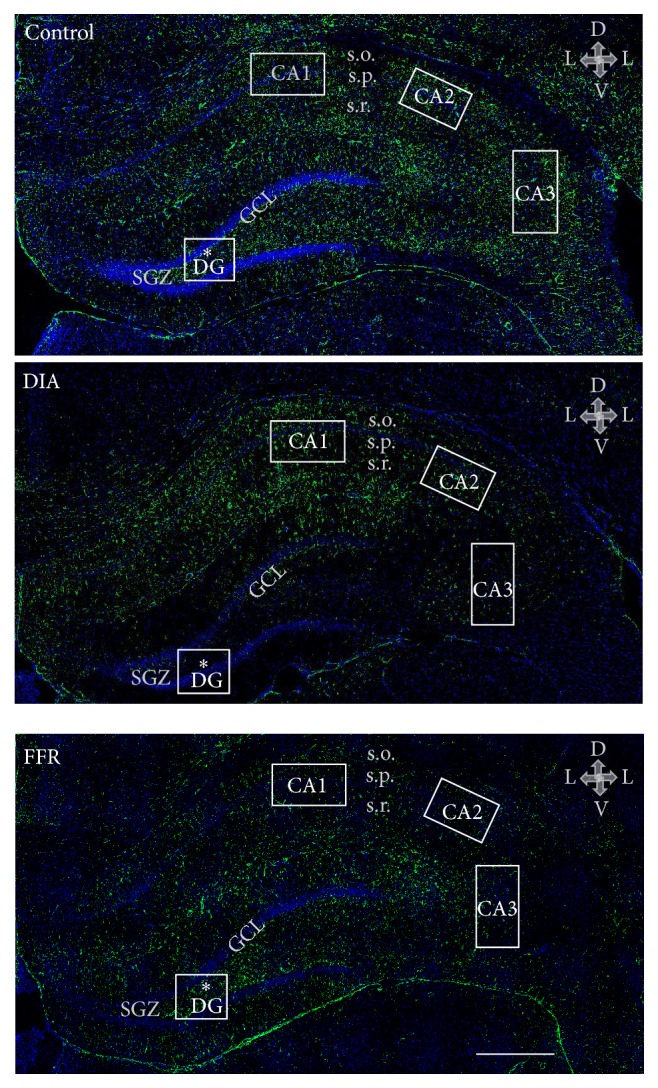
The rat hippocampus. Coronal brain sections from young female rats showing the hippocampus of control, dehydration-induced anorexia (DIA), and forced food-restricted (FFR) rats. The histological sections from the three experimental groups were immunostained for GFAP (green), and nuclei were labeled with DAPI (blue). The hippocampal regions for this study included the cornus ammonis 1–3 (CA1, CA2, and CA3) and the dentate gyrus (DG). Nuclear and astrocyte densities were estimated in the stratum oriens (s.o.) and stratum radiatum (s.r.) of CA1, CA2, and CA3, while the hilus region (*∗*) was studied for the DG (squares). Other regions are indicated for anatomical reference: stratum pyramidale (s.p.), granular cell layer (GCL), and subgranular zone (SGZ). Arrows show anatomical orientation: dorsal (D), ventral (V), and lateral (L). Scale bars correspond to 200 *μ*m.

Mounted coronal sections containing the hippocampus were photographed with a digital camera (Photometrics Cool Snap FX, USA) attached to a Nikon microscope (Nikon Eclipse E600, Tokyo, Japan) and analyzed using IMAGE J version 1.41 (NIH, Bethesda MD, USA). A Zeiss LSM 780 Meta Confocal Microscope (Zeiss, Göttingen, Germany) was used for confocal images with Alexa 594 (excitation/emission wavelength 590/617 nm) and DAPI (excitation/emission wavelength 350/460 nm) (Figure 1).

### 2.5. Cell Counting

GFAP-immunolabeled cells were counted and contrasted with the number of DAPI-labeled nuclei for each subfield of the hippocampus: CA1, CA2, CA3, and dentate gyrus (DG). A total of 2-3 aleatory fields in each of the three tissue sections selected from each of 6–8 animals per group were used for cell counting and “*n*” refers to the total aleatory fields used from all the tissue sections from all the animals of each experimental group. A test square grid of 100 × 100 *μ*m (0.01 mm^2^) was used to estimate the number of DAPI-stained nuclei (equivalent to the total number of cells) and GFAP-labeled cells corresponding to astrocytes, manually and with Cell Profiler [13, 15]. Only process-bearing cells showing their soma in the plane of the analyzed area were counted, and their density was estimated (number per mm^2^). Six to eight randomly chosen fields located within each of the analyzed hippocampal subfields were counted by progressive displacement of a test square grid. The astrocytes/nuclei ratio was calculated by dividing the number of GFAP+ cells by the total number of nuclei labeled with DAPI. GFAP+ cells were identified as resting (with small somata bearing long, thin, and ramified processes) or reactive (with retraction of processes to a length shorter than the diameter of the somata) [16]. The number of reactive astrocytes was estimated for all the experimental groups and normalized with the control.

All the data corresponding to each treatment and region were pooled and are presented as the mean ± standard error of the mean (SEM). Statistical analysis of data was performed using a one-way ANOVA followed by a Bonferroni posttest with Origin 7.0 software. *p* < 0.05 was considered statistically significant.

### 2.6. Western Blot

The hippocampus was dissected out as previously described [17]. The hippocampi from three different rats for each experimental group (control, DIA, and FFR) were homogenized in ice cold lysis buffer (200 mM glycine, 150 mM NaCl, 50 mM EGTA, 50 mM EDTA, and 300 mM sucrose) with a protease inhibitor (Sigma-Aldrich, USA). The homogenate was centrifuged at 10,000 ×g for 15 minutes at 4°C, and the supernatants were aliquoted and stored at −80°C. Concentration of each protein sample was estimated by Bradford's method [18]. An equal concentration of protein (30 *μ*g) was resolved by 10% polyacrylamide gel electrophoresis (PAGE). The proteins were transferred electrophoretically to PVDF membrane; then the membranes were blocked with 5% nonfat dry milk in TBS-T for 3 h at room temperature (RT). Membranes were incubated at 4°C overnight with one of the following primary antibodies: (a) polyclonal rabbit anti-GFAP antibody (dilution 1 : 2000, DakoCytomation, Fort Collins, CO, USA); (b) polyclonal rabbit anti-vimentin (dilution 1 : 2500, Cell Signaling, Danvers, MA, USA); (c) monoclonal mouse anti-nestin (dilution 1 : 5000, BD Biosciences, San José, CA, USA); and (d) polyclonal goat anti-actin (dilution 1 : 2500, Santacruz, Dallas, TX, USA). The membranes were washed, and bound antibodies were detected by incubating for 3 h with either the goat anti-rabbit alkaline phosphatase conjugated antibody (dilution 1 : 2500; Santacruz, Dallas, TX); goat anti-mouse alkaline phosphatase conjugated antibody (dilution 1 : 2500; Santacruz, Dallas, TX); or rabbit anti-goat alkaline phosphatase conjugated antibody (dilution 1 : 2500; Santacruz, Dallas, TX). Then the membrane was washed with TBS-T and the alkaline phosphatase activity was detected with BCIP/NBT AP-conjugate substrate reaction Kit (Bio-Rad, USA). The intensity of each band was measured using Image Lab 3.0 software.

## 3. Results

The effects of DIA and FFR on the density of nuclei and astrocytes were estimated for the following hippocampal regions: CA1, CA2, CA3, and dentate gyrus (DG) (Figure 1). All observations included the stratum radiatum and stratum oriens, where astrocytes are preferentially located, while for the DG the observations were performed in the hilus (Figure 1).

### 3.1. Anorexia Had No Effect on GFAP+ Cell Density in CA1

**Figure 2:**
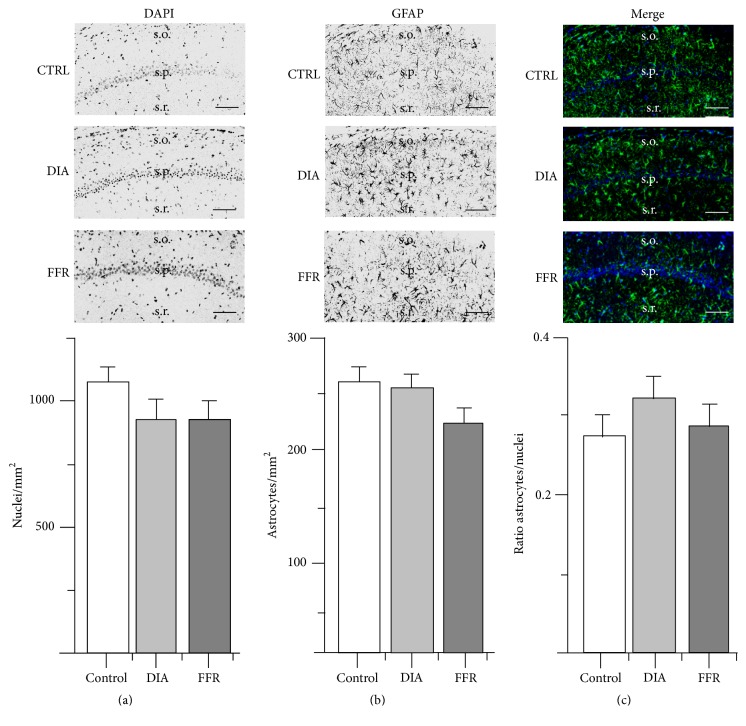
Effect of dehydration-induced anorexia (DIA) on astrocyte density of hippocampal region CA1. Coronal sections of the hippocampus show nuclei labeled with DAPI (a) or immunostained with GFAP (b). The overlay of DAPI and GFAP is shown for control, dehydration-induced anorexia (DIA), and forced food restricted (FFR) experimental groups (c). The densities of nuclei or GFAP+ cells as well as the ratio of astrocyte/nuclei did not differ significantly among experimental groups (*p* = 0.075; *p* = 0.172; *p* = 0.417 for the control, DIA, and FFR groups, respectively; see Table 1). Nuclear and astrocyte densities were estimated in the stratum oriens (s.o.) and stratum radiatum (s.r.); stratum pyramidale (s.p.) is indicated as a reference. Scale bar = 50 *μ*m. Data are mean ± SEM.

The hippocampal region CA1 showed no significant change in nuclear density between the control (1099 ± 52 nuclei/mm^2^; *n* = 62), DIA (924 ± 67 nuclei/mm^2^; *n* = 56), and FFR groups (932 ± 68 nuclei/mm^2^; *n* = 52) (*p* = 0.075) (Figure 1 and Table 1). Additionally, no significant changes were observed for astrocyte density between the control (257 ± 14 nuclei/mm^2^; *n* = 82), DIA (253 ± 14 nuclei/mm^2^; *n* = 72), and FFR groups (221 ± 15 nuclei/mm^2^; *n* = 66) (*p* = 0.172) (Figure 2 and Table 1). We also determined the astrocyte/nuclei ratio by estimating the GFAP+ cells from the total nuclei. The astrocyte/nuclei ratio for the control group was 0.277 ± 0.025 (*n* = 62), and it was not significantly different for the DIA and FFR groups (0.324 ± 0.025; *n* = 56 and 0.286 ± 0.029; *n* = 52; *p* = 0.417) (Figure 2 and Table 1).

### 3.2. Anorexia Reduced GFAP+ Cell Density in CA2

**Figure 3:**
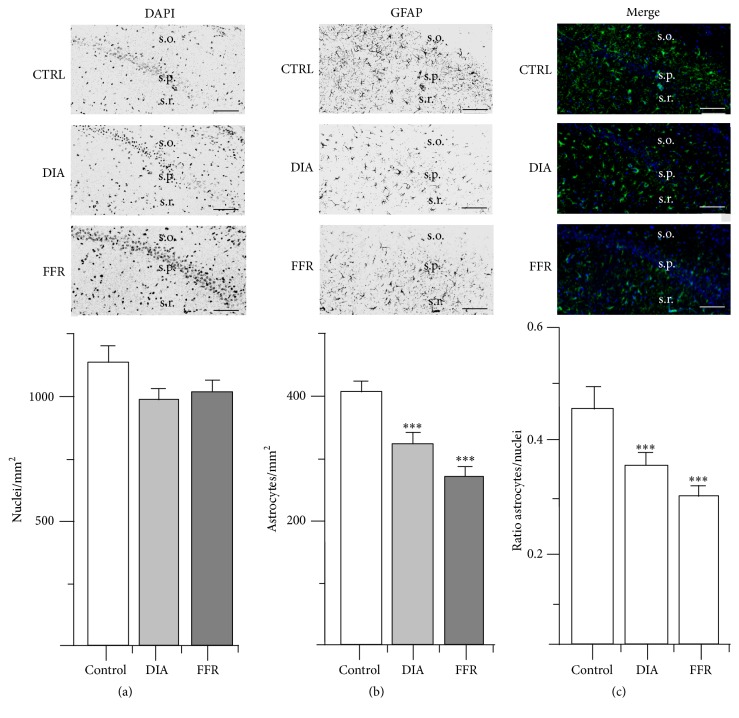
Effect of dehydration-induced anorexia (DIA) on astrocyte density of hippocampal region CA2. Coronal sections of the hippocampus show DAPI (a) or GFAP immunostaining (b). The overlay of DAPI and GFAP is shown for control, dehydration-induced anorexia (DIA), and forced-food restricted (FFR) experimental groups (c). The density of the nuclei was similar in all experimental groups (*p* = 0.467). However, a significant reduction of GFAP+ cells was observed for the DIA and FFR groups (*p* < 0.001). This reduction was consistently observed in the astrocyte/nuclei ratio (*p* = 0.008) (see Table 1). Nuclear and astrocyte densities were estimated in the stratum oriens (s.o.) and stratum radiatum (s.r.); stratum pyramidale (s.p.) is indicated as an anatomical reference. Scale bar = 50 *μ*m. Data are expressed as mean ± SEM.

Nuclear density in CA2 was similar for the control (1125 ± 101 nuclei/mm^2^; *n* = 40), DIA (1007 ± 51 nuclei/mm^2^; *n* = 40), and FFR groups (1022 ± 56 nuclei/mm^2^; *n* = 36) (*p* = 0.467) (Figure 3; Table 1). In contrast, the astrocyte density estimated for the control group (405 ± 19 astrocytes/mm^2^; *n* = 40) was significantly reduced for the DIA (323 ± 18 astrocytes/mm^2^; *n* = 40) and FFR groups (267 ± 19 astrocyte/mm^2^; *n* = 36) (*p* < 0.001) (Figure 3; Table 1). Accordingly, the astrocyte/nuclei ratio estimated for the control group (0.447 ± 0.053; *n* = 40) was also significantly reduced for the DIA (0.345 ± 0.021; *n* = 40) and FFR groups (0.282 ± 0.024; *n* = 36) (*p* = 0.008) (Figure 3; Table 1).

### 3.3. Anorexia Reduced GFAP+ Cell Density in CA3

**Figure 4:**
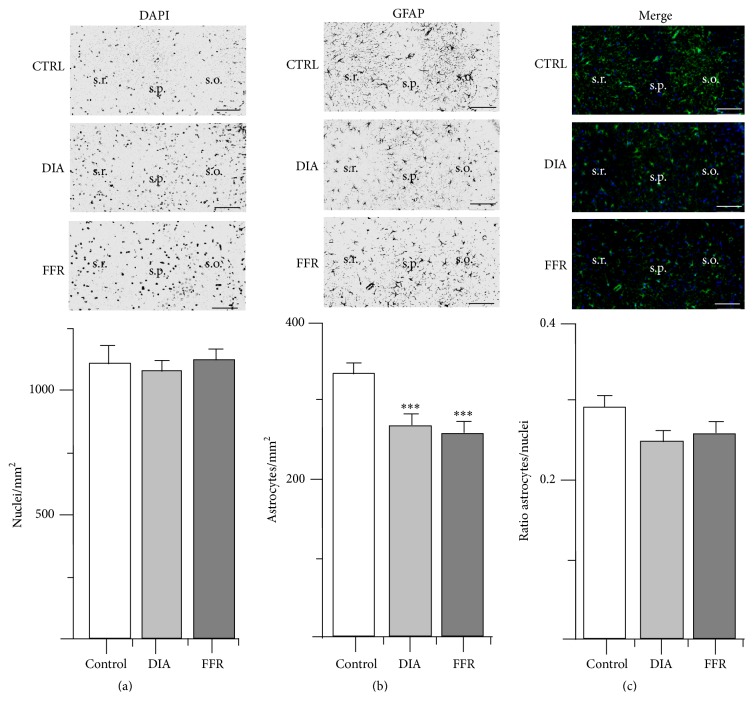
Effect of dehydration-induced anorexia (DIA) on astrocyte density of hippocampal region CA3. Coronal sections of the hippocampus show DAPI (a) or GFAP immunostaining (b). The overlay of DAPI and GFAP is shown for control, dehydration-induced anorexia (DIA), and forced-food restricted (FFR) experimental groups (c). Neither the density of nuclei nor the astrocyte/nuclei ratio differed significantly among groups (*p* = 0.931 and *p* = 0.347, resp.). However, a significant reduction of GFAP+ cell density was observed (*p* < 0.001) (see Table 1). Nuclear and astrocyte densities were estimated in the stratum oriens (s.o.) and stratum radiatum (s.r.); stratum pyramidale (s.p.) is indicated as an anatomical reference. Scale bar = 50 *μ*m. Data are expressed as mean ± SEM.

The nuclear density of CA3 did not show significant changes between the control (1288 ± 43 nuclei/mm^2^; *n* = 62), DIA (1084 ± 41 nuclei/mm^2^; *n* = 56), and FFR groups (1117 ± 46 nuclei/mm^2^; *n* = 52) (*p* = 0.001) (Figure 4; Table 1). However, the astrocyte density of the control group (332 ± 19 astrocytes/mm^2^; *n* = 82) was significantly reduced for the DIA (269 ± 15 astrocytes/mm^2^; *n* = 72) and FFR groups (258 ± 15 astrocyte/mm^2^; *n* = 66) (*p* < 0.001) (Figure 4; Table 1). However, the astrocyte/nuclei ratio estimated for the control group (0.292 ± 0.016; *n* = 62) was not significantly reduced in the DIA (0.262 ± 0.016; *n* = 56) and FFR groups (0.264 ± 0.016; *n* = 52) (*p* = 0.347) (Figure 4; Table 1).

### 3.4. Anorexia Reduced GFAP+ Cell Density in DG

**Figure 5:**
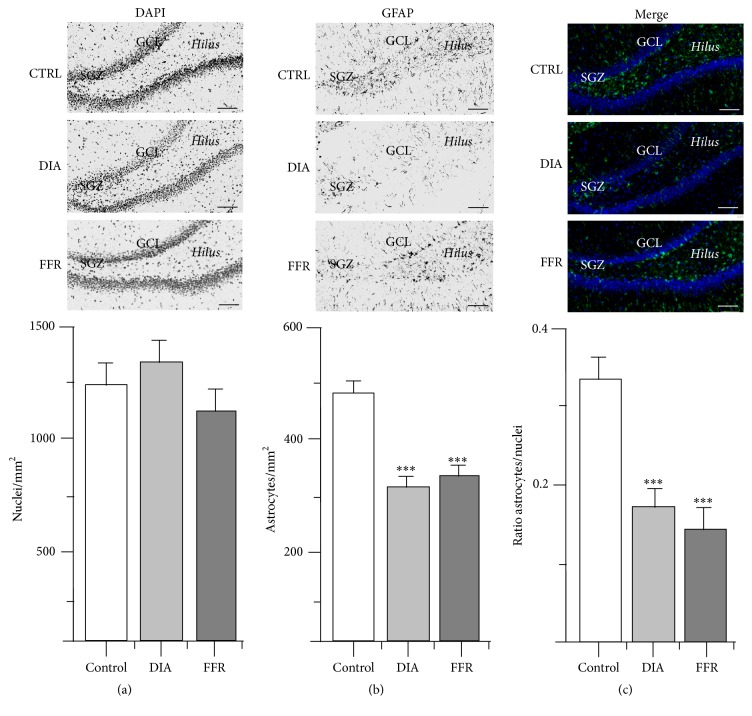
Effect of dehydration-induced anorexia (DIA) on astrocyte density of hippocampal dentate gyrus (DG). Coronal sections of the hippocampus show DAPI (a) or GFAP immunostaining (b). The overlay of DAPI and GFAP is also shown for the control, DIA, and forced food-restricted (FFR) groups (c). The density of nuclei did not differ significantly among experimental groups (*p* = 0.398). However, a significant reduction was observed for GFAP+ cell density and the astrocyte/nuclei ratio (*p* < 0.001 and *p* < 0.001, resp.) (see Table 1). Nuclear and astrocyte densities were estimated in the hilus; subgranular zone (SGZ) and granular cell layer (GCL) are indicated as an anatomical reference. Scale bar = 50 *μ*m. Data are expressed as mean ± SEM.

The nuclear density of the dentate gyrus did not differ significantly for the control (1433 ± 110 nuclei/mm^2^; *n* = 21), DIA (1535 ± 142 nuclei/mm^2^; *n* = 20), and FFR groups (1268 ± 161 nuclei/mm^2^; *n* = 19) (*p* = 0.398) (Figure 5; Table 1). The astrocyte density of the control group (476 ± 28 astrocytes/mm^2^; *n* = 41) was significantly reduced for the DIA (294 ± 124 astrocytes/mm^2^; *n* = 36) and FFR groups (321 ± 26 astrocyte/mm^2^; *n* = 32) (*p* < 0.001) (Figure 5; Table 1). Accordingly, the astrocyte/nuclei ratio estimated for the control group (0.334 ± 0.031; *n* = 21) was significantly reduced for the DIA (0.173 ± 0.022; *n* = 20) and FFR groups (0.139 ± 0.032; *n* = 19) (*p* < 0.001) (Figure 5; Table 1).

#### 3.4.1. Expression of Intermediate Filaments Is Affected by Anorexia

**Figure 6:**
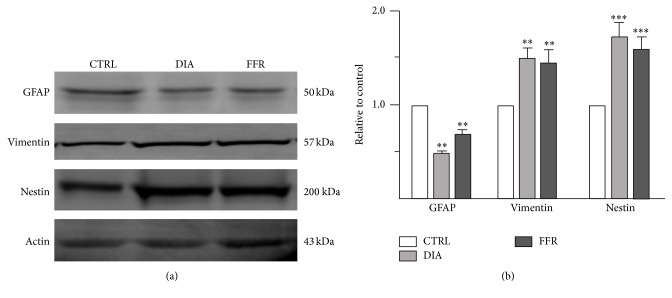
Effect of anorexia on astrocyte intermediate filaments expression after the fifth day. (a) Representative Western blot showing the expression of GFAP, vimentin, and nestin for control, DIA, and FFR experimental groups; actin was used as an internal control. (b) The band intensity of DIA and FFR groups was normalized to their respective controls. Data were shown as mean ± SEM. Significant differences were considered as ^*∗∗*^ *p* < 0.01; ^*∗∗∗*^ *p* < 0.001.

The expression of intermediate filaments of astrocytes was tested by Western blot in the control, DIA, and FFR experimental groups (Figure 6). The expression of GFAP was significantly reduced by DIA (0.48 ± 0.02; *n* = 3; *p* = 0.003) and FFR (0.67 ± 0.04; *n* = 3; *p* = 0.006) when compared to the normalized control; no significant differences were observed between DIA and FFR groups (*p* = 0.668) (Figures 6(a) and 6(b)). The opposite effect was observed for the expression of vimentin and nestin. The expression of vimentin was significantly increased by DIA (1.51 ± 0.08; *n* = 3; *p* = 0.009) and FFR (1.46 ± 0.11; *n* = 3; *p* = 0.008), when compared to the normalized control; no significant differences were observed between DIA and FFR groups (*p* = 0.896) (Figures 6(a) and 6(b)). Finally, the expression of nestin was significantly increased by DIA (1.74 ± 0.1; *n* = 3; *p* = 0.004) and FFR (1.61 ± 0.12; *n* = 3; *p* = 0.001) when compared to the normalized control; no significant differences were observed between DIA and FFR groups (*p* = 0.468) (Figures 6(a) and 6(b)).

#### 3.4.2. Anorexia Decreases GFAP Expression after the Third Day

**Figure 7:**
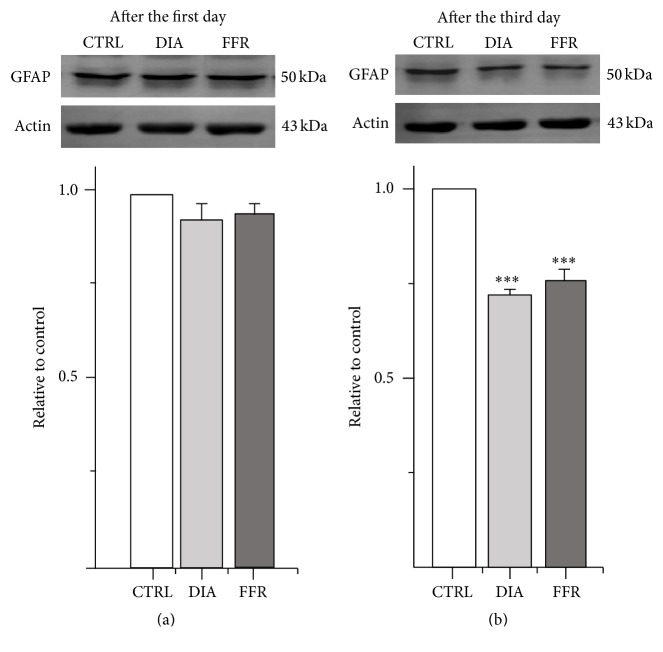
GFAP expression is reduced after the third day. (a, b) Representative Western blot showing the expression of GFAP after the first (a) and third day (b) of anorexia; actin was used as an internal control. The band intensity of DIA and FFR groups was normalized to their respective controls ((a, b) under the Western blots). Data were shown as ± SEM. Significant differences were considered as ^*∗∗∗*^ *p* < 0.001.

Our results showed a reduced GFAP+ cell density in the hippocampus that correlated with a lower expression of the protein. Nevertheless, GFAP expression may be affected earlier than five days. Thus, the expression of GFAP was tested by Western blot after one and three days for control, DIA, or FFR experimental groups (Figures 7(a) and 7(b)). The results showed that the expression of GFAP was unaffected after the first day for DIA (0.92 ± 0.04) and FFR (0.94 ± 0.03), when compared to the normalized control (*p* > 0.1). However, GFAP expression was significantly reduced after the third day for DIA (0.72 ± 0.01) and FFR (0.75 ± 0.04), when compared to the normalized control (*p* < 0.001) (Figures 7(a) and 7(b)).

### 3.5. Anorexia Increases Reactive Astrocytes

**Figure 8:**
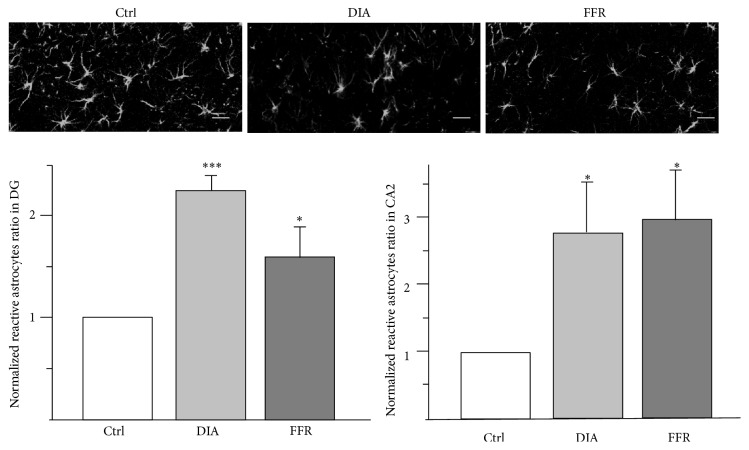
Anorexia increases the density of reactive astrocytes. Representative images of GFAP+ cells in the dentate gyrus (DG) for control (Ctrl), dehydration-induced anorexia (DIA), and forced food restriction (FFR) groups. The density of reactive astrocytes (cells with processes shorter than the diameter of the somata) was estimated for all the experimental groups and normalized with their respective controls for DG and CA2. Data were shown as mean ± SEM. Significant differences were considered as ^*∗*^ *p* < 0.05; ^*∗∗∗*^ *p* < 0.001.

The reactive astrocytes were estimated in CA2 and DG, because astrocyte-nuclei ratio was significantly reduced in these regions. Reactive astrocytes were defined as GFAP+ cells with processes shorter than the diameter of the somata [16]. The number of reactive astrocytes was estimated for all the experimental groups and normalized with their respective controls (see Section 2.5). In CA2, reactive astrocytes increased 2.76 ± 0.7 (*n* = 4; *p* = 0.047) for DIA and 2.92 ± 0.75 (*n* = 4; *p* = 0.032) for FFR, while for dentate gyrus, reactive astrocytes increased 2.25 ± 0.15 (*n* = 4; *p* < 0.0001) for DIA and 1.61 ± 0.29 (*n* = 4; *p* = 0.034) for FFR (Figure 8).

## 4. Discussion

Anorexia nervosa is common among female adolescents and has one of the highest mortality rates within psychiatric disorders (10–20%), even surpassing depression [19–21]. During puberty, mood swings, and anxiety are common due to a sudden rise of hormones which affects the hippocampus [11, 12, 22]. Moreover, in the activity-based anorexia model (ABA), structural and functional alterations have been observed in the hippocampus, accompanied by anxiety-like behavior [1, 11, 12, 23]. However, the effect of anorexia on astrocyte density has been barely explored. Indeed, previous studies in the rat corpus callosum reported that cell proliferation was reduced with the ABA model, while astrocyte density was regionally reduced in the DIA model [1, 13]. The ABA study also reported a reduced hippocampal cell proliferation in the dentate gyrus [1].

Results from the present study show that anorexia reduced astrocyte density in CA2, CA3, and DG, while no significant changes were observed in CA1. Accordingly, anorexia mutant mice (*anx/anx*) showed lower expression of c-Fos in the dentate gyrus and CA3 region of the hippocampus, while no significant differences were found in CA1 [24]. On the other hand, the decrease in GFAP+ cell density resulted in a reduced expression of GFAP protein. Astrocytes express three types of intermediate filament proteins: GFAP, vimentin, and nestin [25]. Nestin and vimentin are the main intermediate filaments in immature astrocytes, whereas maturing and adult astrocytes contain vimentin and GFAP [25]. Interestingly, anorexia increased vimentin and nestin expression resembling reactive astrocytes observed after injury [26, 27]. Accordingly, GFAP+ cells deficit may be replaced by vimentin+/nestin+ cells in anorexia. In support of this hypothesis, nestin reexpression has been observed following experimental hippocampal lesions [25, 28, 29] and also in human pathology such as multiple sclerosis [30]. Moreover, reactive astrocytes were increased by anorexia and forced food restriction, indicating that GFAP+ cells react to severe caloric restriction [31]. Another factor to consider is that female animals subjected to anorexia or chronic stress stop their ovarian cycles, ending their exposure to hormones that may affect morphology and astrocyte density [32, 33]. Moreover, hippocampal gliogenesis, but not neurogenesis, is reduced in the ABA model [1]. Thus, astrocytes possess different levels of differentiation and maturation and they can switch between these states even in the adult brain [26]. Likewise, astrocytes secrete gliotransmitters and growth factors like BDNF, required for synaptic plasticity, synaptogenesis, and neurogenesis [34, 35]. Thus, a reduced density of GFAP+ cells may affect hippocampal gliotransmission of d-serine, a gliotransmitter necessary for neuronal NMDA-mediated transmission and long term potentiation [34]. Growth factors such as BDNF are also required for long term synaptic plasticity of dendrites in the adult hippocampus [35]; however, decreased branching of dendrites was reported in an ABA model, particularly in the stratum radiatum [11, 12]. Accordingly, our results showed that GFAP+ cell density and GFAP expression are decreased by anorexia. Similar results were reported in a rat model of depression, where GFAP expression was reduced [36]. Thus, we conclude that GFAP+ cells and intermediate filament expression are affected by anorexia. Our results add new information about the structural changes observed in the hippocampus of murine models of anorexia.

## Supplementary Material

The experimental protocol was conducted for five days and body weight and solid food intake were recorded daily at noon for each experimental group, over five days. The FFR group received the same amount of food as that ingested by DIA animals.

## Figures and Tables

**Table 1:** Regional changes in astrocyte density in DIA and FFR.

Hippocampal region	Control	DIA	FFR	*p*
Nuclei				
CA1	1099 ± 52 (*n* = 62)	924 ± 67 (*n* = 56)	932 ± 68 (*n* = 52)	0.075
CA2	1125 ± 101 (*n* = 40)	1007 ± 51 (*n* = 40)	1022 ± 56 (*n* = 36)	0.467
CA3	1288 ± 43 (*n* = 62)	1084 ± 41^*∗∗∗*^ (*n* = 56)	1117 ± 46^*∗∗∗*^ (*n* = 52)	0.001
DG/SGZ	1433 ± 110 (*n* = 21)	1535 ± 142 (*n* = 20)	1268 ± 161 (*n* = 19)	0.398

Astrocytes				
CA1	257 ± 14 (*n* = 82)	253 ± 14 (*n* = 72)	221 ± 15 (*n* = 66)	0.172
CA2	405 ± 19 (*n* = 40)	323 ± 18^*∗∗∗*^ (*n* = 40)	267 ± 19^*∗∗∗*^ (*n* = 36)	<0.001
CA3	332 ± 14 (*n* = 82)	269 ± 15^*∗∗∗*^ (*n* = 72)	258 ± 15^*∗∗∗*^ (*n* = 66)	<0.001
DG/SGZ	476 ± 28 (*n* = 41)	294 ± 24^*∗∗∗*^ (*n* = 36)	321 ± 26^*∗∗∗*^ (*n* = 32)	<0.001

Astrocytes/nuclei ratio				
CA1	0.277 ± 0.025 (*n* = 62)	0.324 ± 0.025 (*n* = 56)	0.286 ± 0.029 (*n* = 52)	0.417
CA2	0.447 ± 0.053 (*n* = 40)	0.345 ± 0.021^*∗∗∗*^ (*n* = 40)	0.282 ± 0.024^*∗∗∗*^ (*n* = 36)	0.008
CA3	0.292 ± 0.016 (*n* = 62)	0.262 ± 0.016 (*n* = 56)	0.264 ± 0.016 (*n* = 52)	0.347
DG/SGZ	0.334 ± 0.031 (*n* = 21)	0.173 ± 0.022^*∗∗∗*^ (*n* = 20)	0.139 ± 0.032^*∗∗∗*^ (*n* = 19)	<0.001
