# Supplementary figures and images for: Anorexia Reduces GFAP+ Cell Density in the Rat Hippocampus
Source: Neural Plast. 2016 Aug 7;2016:2426413. doi: 10.1155/2016/2426413 (PMC4992534; doi:10.1155/2016/2426413)

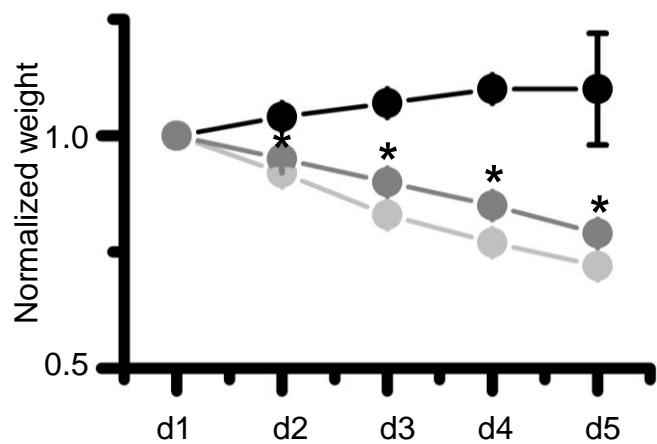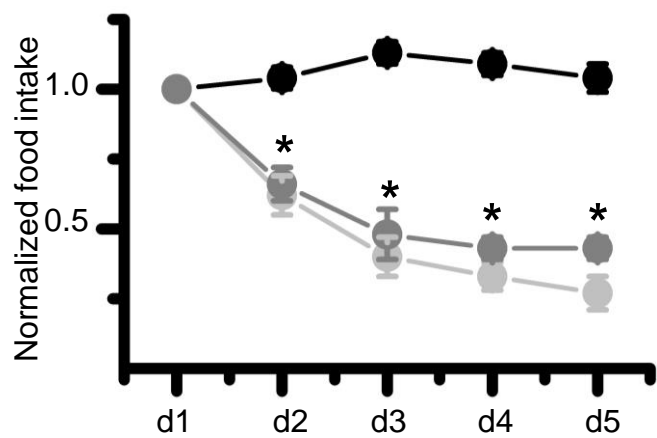

Supplemental Fig. 1

Supplement: Supplementary file 1 — The experimental protocol was conducted for five days and body weight and solid food intake were recorded daily at noon for each experimental group, over five days. The FFR group received the same amount of food as that ingested by DIA animals. [file 2426413.f1.pdf]
